# Colorimetric Detection of microRNA-378 Based on Y-Shaped Structure Formed by Gold Nanoparticles and Catalytic Hairpin Self-Assembly

**DOI:** 10.3390/bios15050319

**Published:** 2025-05-15

**Authors:** Yahui Gao, Jinru Pan, Bingyuan Fan, Shan Wang, Qian Wang, Wanru Liu, Fang Hu, Wei Meng

**Affiliations:** Key Laboratory of Biomedical Functional Materials, School of Sciences, China Pharmaceutical University, Nanjing 211198, China; 15224768389@163.com (Y.G.); pjinru@126.com (J.P.); 15380917330@163.com (B.F.); wangshan19992021@163.com (S.W.); wq106996802@163.com (Q.W.); liuwr12@163.com (W.L.)

**Keywords:** gold nanoparticles, catalytic hairpin assembly, MicroRNA, colorimetry

## Abstract

The timely and accurate detection of cancer is crucial for preventing disease progression and for the early treatment of confirmed cases. MiRNAs are cancer markers. In this study, a simple miRNA detection method is proposed. Three hairpins were designed based on gold nanoparticles combined with catalytic hairpin assembly nucleic acid amplification technology. The low-pH method was used for rapid coupling, and hairpin H1 was opened by miR-378, triggering the cycle reaction and signal amplification and finally forming a Y-shaped structure, thereby narrowing the distance between gold nanoparticles and achieving colorimetric detection. The absorbance change (A_620_/A_520_) was proportional to the concentration of miR-378 (0.05–5 nM), with a detection limit of 0.05 nM. This method also has an evident detection effect on real samples. HeLa and L-02 cell extracts were analyzed using this method. The former showed no obvious color change, whereas the maximum absorption peak of the latter showed a red shift, and the color changed from red to purple. The minimum number of cells that could be detected using HeLa cells was 500 cells/mL.

## 1. Introduction

MiRNA is a small non-coding RNA with about 19–24 nucleotides that inhibits protein translation by binding to the 3′-UTR of the target mRNA. Because miRNAs are relatively stable and easy to detect, they are often used as noninvasive blood biomarkers [1]. A large amount of evidence indicates that miRNAs play important roles in cancer. Many upregulated and downregulated miRNAs are involved in regulating various cancer processes [2,3]. For example, Liu et al. reported that miR-155 downregulated ErbB2 and inhibited the ErbB2-induced malignant transformation of mammary epithelial cells [4]. Tang et al. found that upregulated miR-21 promotes the progression of lung adenocarcinoma by inhibiting the KIBRA and Hippo signaling pathways [5]. Yang et al. demonstrated that miR-378 promoted cell growth and accelerated the cell cycle by directly downregulating ST7L in HeLa and SiHa cells and inhibiting apoptosis, thus playing the role of an oncogene [6].

Traditional methods for miRNA detection, such as northern blotting (long detection times and large sample sizes) [7,8], have obvious limitations in different aspects and poor microarray specificity [9,10]. Although RT-qPCR has high specificity, primer design is difficult, and pretreatment is complex [11,12]. To enhance the performance of miRNA detection, biosensors utilizing gold nanoparticles (AuNPs) have been progressively developed in recent years. Given the low abundance of miRNAs, detection methods are frequently integrated with nucleic acid amplification techniques to boost their sensitivity. These techniques include the hybridization chain reaction, rolling circle amplification, and catalytic hairpin self-assembly (CHA). Among these, CHA stands out because it does not require enzymes and offers greater flexibility in reaction conditions, such as temperature, making it particularly popular in experimental settings [13,14,15]. Based on CHA, colorimetric detection using functionalized AuNPs is not only easy to perform but the results are also intuitive, which has attracted significant attention in recent years.

Owing to their remarkable attributes, AuNPs have swiftly emerged as cornerstones in diverse scientific and industrial domains [16,17,18]. Biosensors that use the strong surface plasmon resonance and high extinction coefficient of AuNPs to observe their color changes for detection purposes have been repeatedly reported. Huang et al. used one-step competitive analysis to detect thrombin by decomposing oligonucleotide-functionalized AuNP aggregates [19]. Kim et al. successfully detected polystyrene nanoplastics colorimetrically using AuNPs. The color of the solution changed from blue to red as the polystyrene nanoplastic concentration increased [20]. Mollasalehi et al. developed a non-amplified nanocolorimetric biosensor that uses extracellular miRNAs for the simple diagnosis of deadly cancer [21]. Compared with other biosensors, the colorimetric biosensor based on functionalized AuNPs can allow for the direct observation of the color change with the naked eye, without the help of complex instruments and equipment to evaluate the detection results, which is convenient for nonprofessional operation and interpretation. Moreover, the preparation method for AuNPs is relatively simple and results in good chemical and physical stabilities. Surfaces can be modified using various cost-effective chemical methods [22,23].

Cervical and ovarian cancers are particularly common in women and have high mortality rates. Although many advances have been made in research, its incidence continues to increase. Therefore, the sensitive detection of miR-378 is of great significance for the early screening of cancer in women. In this study, a colorimetric biosensor based on AuNPs was developed by combining the characteristics of AuNPs of different colors in different states with the advantages of CHA. Three hairpins containing polyadenine fragments (polyA) were designed. Owing to the strong adsorption of adenine (A) groups on the surface of gold, in the functionalization stage, AuNPs were functionalized using the low-pH method to achieve rapid and efficient coupling. This method significantly reduces time and economic costs. During the detection process, the target sensitively triggers H1 to form a Y-shaped structure. Compared with the traditional double hairpin-assisted CHA reaction, it can shorten the distance of more AuNPs, can change the color of the system before and after the addition of the target, and exhibits higher sensitivity and better specificity. In addition, the applicability of the biosensor to real samples was demonstrated through the detection of cell extracts. In terms of application, the constructed detection method can quickly obtain detection results, which is convenient for nonprofessionals to operate and interpret and is more suitable for ordinary people.

## 2. Experimental Section

### 2.1. Materials and Reagents

All oligonucleotides were synthesized and modified by Shanghai Sangong Biotechnology Co., Ltd. (Shanghai, China) (Table S1). Tetrachloroauric (III) acid tetrahydrate (HAuCl_4_·4H_2_O) was purchased from Shanghai Jiuwo Biotechnology Co., Ltd. (Shanghai, China); trisodium citrate (C_6_H_5_Na_3_O_7_), tris (hydroxymethyl) aminomethane (Tris), and magnesium chloride hexahydrate (MgCl_2_·6H_2_O) were purchased from Sinopharm Chemical Reagents Co., Ltd. (Shanghai, China); hydrochloric acid (HCl), nitric acid (HNO_3_), and sodium chloride (NaCl) were purchased from Nanjing Chemical Reagent Co., Ltd. (Nanjing, China); 50 × TAE, 4SGelred (10,000×) was purchased from Shanghai Shenggong Bioengineering Technology Service Co., Ltd. (Shanghai, China). Unless otherwise specified, all chemicals are of analytical grade.

### 2.2. Apparatus

Absorbance was measured using a Shimadzu UV-1900 UV-visible spectrophotometer (UV-3600, Shimadzu, Japan). The hydrated particle size and zeta potential of nanoparticles were measured using a particle size potentiometer (Nano ZS90, Malvern, UK). The particle size and morphology of AuNPs were observed via transmission electron microscopy (TEM). The gel image was recorded using the Tanon 1600 gel imaging system (Beijing, China).

### 2.3. Preparation and Characterization of AuNPs

According to the method described by our predecessors, the material with a particle size of about 13 nm was prepared using the trisodium citrate reduction method, with slight improvements made on this basis [24]. It should be noted that the glassware used in the experiment should be soaked in aqua regia (HNO_3_:HCl = 1:3) before preparation and then rinsed thoroughly with ultrapure water and dried in an oven. After the preparation, the 1 mM HAuCl4 solution was heated and refluxed to boil. While stirring the solution, the prepared 38.8 mM trisodium citrate solution was quickly added to it, and the reaction solution was observed. The striking color change from pale yellow to dark blue to wine red continued as we heated and boiled the solution for 20 min and then cooled it to room temperature. We then filtered it using a 0.22 μm PES syringe and stored it in the dark at 4 °C. The absorbance of the prepared AuNPs was determined using an ultraviolet-visible spectrometer, and the morphology of the prepared AuNPs was characterized using TEM to determine their particle size and dispersion state.

### 2.4. Functionalization of AuNPs with Oligonucleotides

The hairpin with polyA fragment was designed, and the hairpin with polyAdenine fragment was adhered to the surface of AuNPs using the low-pH method [25]. Hairpins H1, H2, and H3 with polyA fragments were heated at 95 °C for 5 min and cooled to room temperature. The prepared AuNPs and oligonucleotide H1 were placed in an EP tube at a certain ratio, and 0.5 M sodium citrate–hydrochloric acid buffer (pH = 3.3) was added. The mixture was mixed evenly and allowed to stand at room temperature for three minutes. The solution was adjusted to neutral using 0.5 M HEPES solution and centrifuged, and the nanoparticles were retained. The solution was washed three times with 5 mM HEPES buffer to remove the hairpin that did not bind to the AuNPs. Finally, the functionalized gold nanoparticles, AuNPs-H1, were re-suspended. The operations described above were performed using H2 and H3 with the same polyA fragment to obtain gold nanoprobes AuNPs-H2 and AuNPs-H3. The functionalized AuNPs were subsequently collectively referred to as hAuNP. The coupling results were verified using agarose gel electrophoresis, an ultraviolet spectrophotometer, and a particle size potentiometer.

### 2.5. Agarose Gel Electrophoresis

The preparation of hAuNP and the feasibility of detecting miR-378 were confirmed via 4% agarose gel electrophoresis. Agarose powder (1.2 g) was dissolved in 30 mL of 1 × TAE buffer, 2 μL of 4SGelred (10,000×) was added, and the mixture was poured into the mold to cool and form. The prepared samples were mixed with 6 × loading buffer, loaded in different lanes of agarose gel, and run in 1 × TAE running buffer at 100 V voltage for 50 min. The bands of agarose gel were photographed in sunlight with a digital camera, and the gel imaging system and ultraviolet excitation were used for recording.

### 2.6. Colorimetric Detection of miR-378

First, the functionalized AuNPs were prepared according to the method described in Section 2.4. Then, 100 μL of AuNPs-H1, AuNPs-H2, and AuNPs-H3 were added to the EP tube, and a certain concentration of miR-378 was added. The mixture was fully mixed in Tris-HCl buffer and incubated at 25 °C for a period of time. The color change of the sample in the tube was observed, and the absorbance in the range of 300–800 nm was recorded using an ultraviolet spectrophotometer to compare the difference between the target sample and the control sample.

### 2.7. Extraction of Intracellular RNA

HeLa cells were obtained from the Beyotime Institute of Biotechnology (Shanghai, China), and L02 cells were obtained from the Cell Bank of the Chinese Academy of Sciences. The HeLa cells in the exponential growth phase were digested, counted, and centrifuged to obtain the cell precipitate. RNAex was added to the precipitate and blown evenly. Then, chloroform was added and centrifuged to remove the top layer. After adding isopropanol, the solution was allowed to stand for a moment before centrifuging and discarding the supernatant. Pre-cooled ethanol was added to it, the RNA precipitation was washed, the lid was opened and dried, and finally, DEPC water was added to dissolve it.

## 3. Results and Discussion

### 3.1. Design and Working Principle of Nanoprobes

**Scheme 1:**
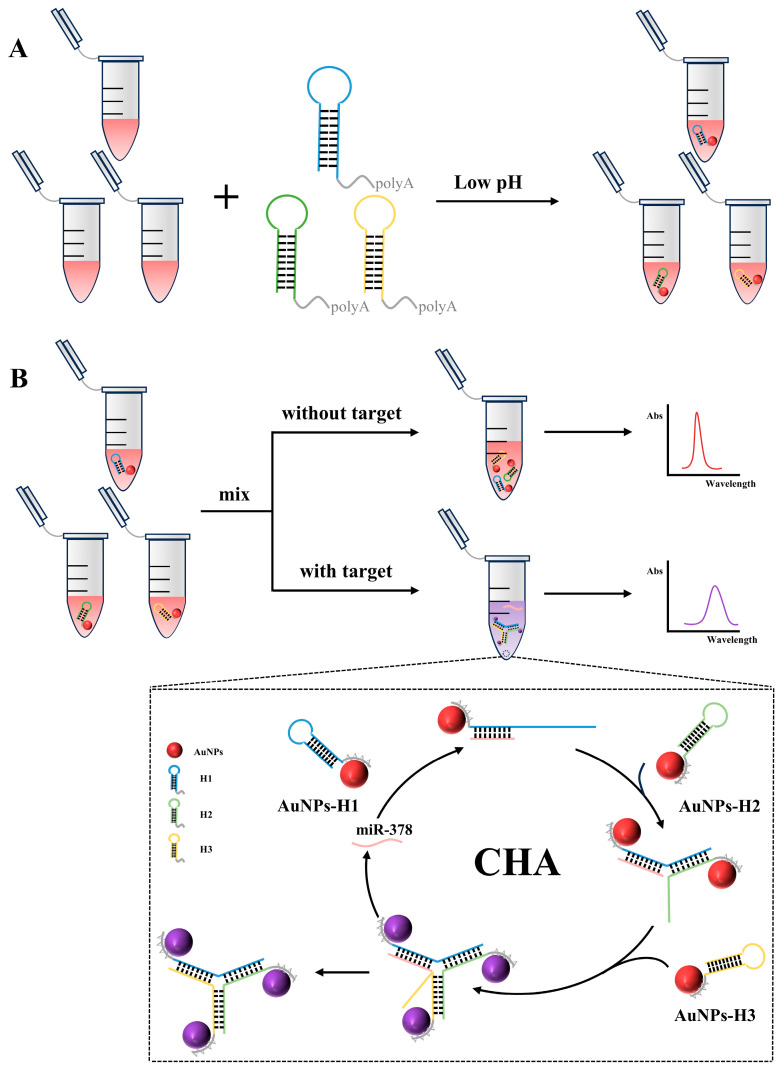
Schematic diagram of the colorimetric detection of miR-378 based on AuNPs and catalytic hairpin self-assembly to form Y-shaped structures. (**A**) Functionalized gold nanoparticles by low pH method. (**B**) Colorimetric detection of miR-378. Purple Ball showed that the complementary pairing of hairpins pulls the gold nanoparticles closer together, creating the SPR effect.

The principle of the proposed miRNA detection strategy is illustrated in Scheme 1. Hairpins H1, H2, and H3 are composed of two domains: the domain involved in hybridization and the polyadenine anchor block. As shown in Scheme 1A, under low pH conditions, the polyadenine moieties of hairpins H1, H2, and H3 can be coupled with AuNPs to obtain functionalized AuNPs. Due to the adsorption of anions, such as citric acid, during the synthesis process, the surface of gold nanoparticles is negatively charged. Under acidic conditions, the concentration of hydrogen ions in the solution increases, and these hydrogen ions will react with the negative charge on the surface of gold nanoparticles, resulting in a significant reduction or even disappearance of the surface. This charge neutralization effect weakens the electrostatic repulsion between gold nanoparticles and negatively charged molecules such as nucleic acids, which leads to the dominance of the Johannes Diderik van der Waals force and the modification of AuNPs. The part of the H1 that is involved in hybridization is complementary to miR-378. The hybridization process is illustrated in Scheme 1B. In the presence of miR-378, the hairpin H1 opens to form an H1-T double chain. At the same time, the exposed single strand can complement the hairpin of H2, thus opening the hairpin H2 to form the H1-H2-T complex. At this point, a single strand exposed by H2 can complement H3 to form H1-H2-H3-T. Because the structure of H1-H2-H3 is more stable than that of H1-H2-H3-T, the single chain exposed to H3 can complement H1 and replace miR-378. The replaced miR-378 is free in the reaction system, which can trigger the opening of the other hairpin, H1, again and participate in the formation of the next round of H1-H2-H3. This structure shortens the distance between AuNPs, promotes their aggregation, and causes color changes. The replaced miR-378 was used to open another hairpin, H1, to facilitate the next round of reactions.

### 3.2. Characterization of AuNPs and hAuNP

**Figure 1:**
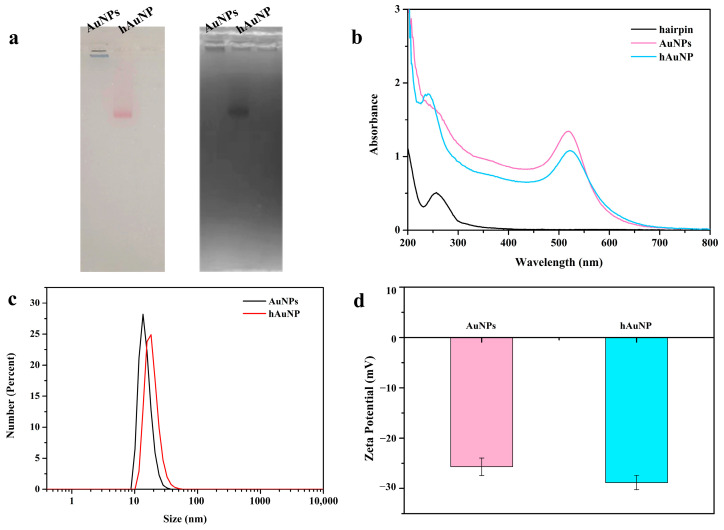
Characterization of gold nanoparticle conjugate AuNPs-H1. (**a**) With the same agarose gel, the results of AuNPs and functionalized AuNPs were recorded using a digital camera and a UV excitation gel recording system, respectively. (**b**) UV characterization of AuNPs and functionalized AuNPs. (**c**) Dynamic light scattering images of AuNPs and functionalized AuNPs. (**d**) Zeta potential of AuNPs and functionalized AuNPs.

To test whether the AuNPs were functionalized at low pH, the samples were subjected to gel electrophoresis (Figure 1a). The sample in lane 1 did not migrate because of the presence of salt ions in the buffer solution, in which bare gold was aggregated and deposited in the upper sample hole. The sample in lane 2 migrated because of the protection provided by the oligonucleotides that stabilized the AuNPs. Under the action of an electric field, the sample migrated into a single sharp band, which also indicated that each AuNP was attached to approximately the same amount of DNA. The same gel also uses ultraviolet light for imaging. In this imaging mode, the AuNPs exhibited a dark band because of the absorption of ultraviolet light. These results prove that the oligonucleotides successfully combined with the AuNPs through polyA. In addition, as shown in Figure 1b, the UV-visible spectrum of AuNPs-H1 showed an absorption peak at 260 nm, which is the characteristic peak of the DNA base, indicating that the coupling was successful. In addition, the absorption spectra of hAuNPs and AuNPs showed similar characteristics. The absorption peak of the hAuNPs was at 522 nm and that of the AuNPs was at 519 nm. Compared with the unmodified AuNPs, a small red shift was observed, indicating that the coupling of hairpin DNA did not change the absorption characteristics of the AuNPs. Dynamic light scattering showed that the hydrodynamic size of the AuNPs was 13.54 nm (Figure 1c). After DNA modification, owing to the presence of the DNA layer, the size increased to approximately 18.17 nm, and no large aggregates were detected, which is consistent with the UV-Vis data. To further prove the successful preparation of the gold nanoprobes, their zeta potentials were measured (Figure 1d). The zeta potential of the probe was lower than that of the AuNPs because of the negative potential of the DNA chain. The change in zeta potential further confirmed the successful preparation of the nanoprobes.

### 3.3. Feasibility of the Fabricated Biosensing Platform

**Figure 2:**
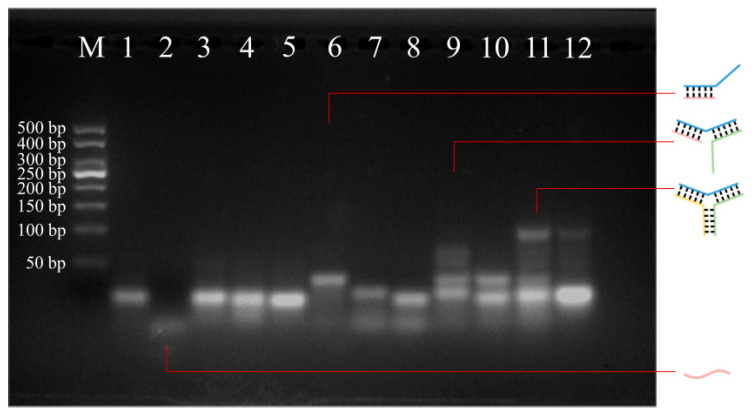
Agarose gel electrophoresis used to verify the feasibility of hairpin hybridization. M: Marker; Lane 1: H1; Lane 2: miR-378; Lane 3: H1 + H2; Lane 4: H2 + H3; Lane 5: H1 + H3; Lane 6: H1 + miR-378; Lane 7: H2 + miR-378; Lane 8: H3 + miR-378; Lane 9: H1 + H2 + miR-378; Lane 10: H1 + H3 + miR-378; Lane 11: H1 + H2 + H3 + miR-378; and Lane 12: H1 + H2 + H3.

The feasibility of hybridization in the presence of miR-378 was verified using agarose gel electrophoresis. As shown in Figure 2, the bands of lanes 3–5 are the same as the migration position of lane 1, indicating that the two hairpins will not hybridize with each other. Lane 6 showed a band that migrated slower than lane 1, and the band at the location of miR-378 was significantly lighter than that of lane 2, indicating that miR-378 opened hairpin H1 and hybridized to form the H1-T complex. Lanes 7 and 8 indicate that miR-378 could not open hairpins H2 and H3. The migration of lane 9 was slower than that of lane 6, indicating formation of the H1-H2-T complex. Similarly to lane 9, there was no H1-H2-T complex in lane 10, indicating that H1 and T could not open the hairpin H3 in the absence of H2. Compared to lane 9, lane 11 showed a slower migration rate in the presence of miR-378, indicating the formation of a Y-shaped structure. Compared with lane 11, the faster migration band in lane 12 was brighter, indicating that there was almost no reaction without miR-378. This result confirms that the hairpin designed for hybridization was feasible. The feasibility of the colorimetric detection was verified using an ultraviolet spectrophotometer. In addition, the delta G of H1-miR-378, H1-H2, H2-H3, and H3-H1 were calculated using the OligoAnalyzer website, as shown in Table S2.

**Figure 3:**
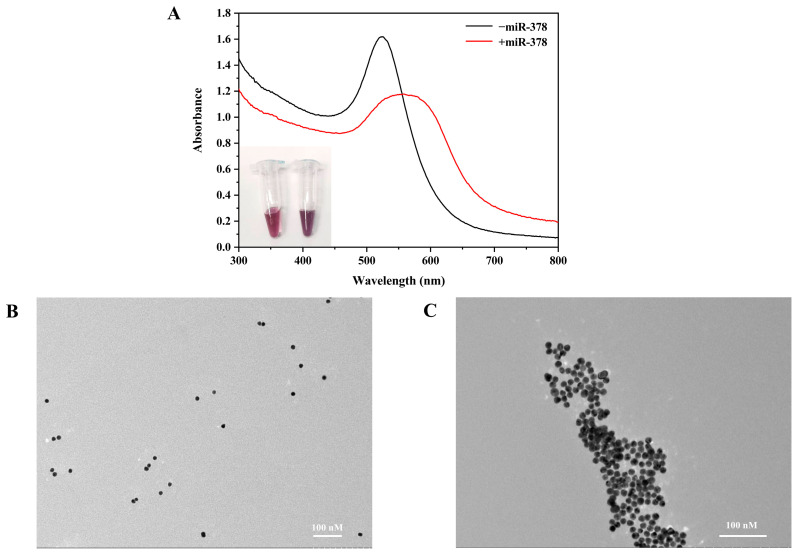
Feasibility verification of colorimetric detection system. (**A**) Verification using UV spectrophotometry. Ultraviolet-visible absorption spectra of miR-378 in the presence and absence of miR-378. (**B**) Transmission electron microscopy characterization before hybridization (the system contained AuNPs-H1, AuNPs-H2, and AuNPs-H3). (**C**) Transmission electron microscopy characterization after hybridization (the system contained AuNPs-H1, AuNPs-H2, AuNPs-H3, and miR-378).

As shown in Figure 3A, when the target miR-378 was present, the UV absorption peak changed dynamically, the UV absorbance decreased, the maximum absorption peak shifted from 522 to 558 nm, and the maximum absorption peak was red-shifted. The color of the reaction solution in the EP tubes changed from red to purple. This indicates that the proposed detection method is feasible. After the feasibility of the detection was preliminarily verified by ultraviolet spectrophotometry, we characterized the changes in the reaction system using transmission electron microscopy. As shown in Figure 3B,C, the nanoprobes were well dispersed in the absence of the target and aggregated in the presence of the target, indicating that the target successfully triggered the CHA reaction and assembled into a Y-shaped structure. This result is consistent with that of the ultraviolet method, which verifies the feasibility of the detection principle.

### 3.4. Optimization of Assay Conditions

To improve detection performance, the experimental conditions, including the length of polyA, pH of the hybridization buffer, and concentration ratio of hairpin to AuNPs, were optimized. Three hairpins with different polyA lengths were designed, with lengths of 10, 15, and 20 bp. The signal-to-noise ratio was used to characterize the detection efficiency of miR-378 at each length. As shown in Figure S2a, A_620_/A_520_ varies with the length of polyA tail. When the polyA15 hairpin was used, it had the highest S/B (S: UV signal in the presence of the target; B: background signal without the DNA target), and the colorimetric effect was better. A possible reason for this is that the shorter polyA on the surface of AuNPs may bring about higher steric hindrance, whereas the longer polyA leads to a lower surface density of the probe on the AuNPs, resulting in a limited signal. In subsequent experiments, hairpins with 15 adenine nucleotides in their tails were used. In addition, the effect of the hybridization buffer on the colorimetric effect was explored. Hybridization buffers with pH values of 6, 7, 8, and 9 were prepared The colorimetric experiment was carried out using the method in Section 2.6 and detected using an ultraviolet spectrophotometer. The results showed that (Figure S2b) compared with other pH values, the Y-type colorimetric biosensor had the best effect on the detection of miR-378 at pH 8. In subsequent experiments, a buffer with a pH of 8 was used as the detection environment.

To further improve detection efficiency, the effect of hairpin concentration on the colorimetric response signal (A_620_/A_520_) was studied. The sticky end of the hairpin structure not only helps the AuNPs maintain a uniform dispersion state but also adjusts the density of the hairpin on the surface of the AuNPs. Therefore, the hairpin concentration is of great significance for the stability of the reaction system, control of the experimental background, and improvement of the signal-to-noise ratio. Concentration ratios (C_hair_/C_AuNPs_) of 50:1, 75:1, 100:1, and 125:1 were selected to determine the optimal concentration ratio. As shown in Figure S2c, as the C_hair_/C_AuNPs_ ratio increased from 50:1 to 100:1, the colorimetric signal gradually increased. When the hairpin concentration continued to increase, the signal did not continue to increase. Therefore, 100:1 was selected as the optimal concentration ratio for the subsequent experiments.

### 3.5. Selectivity of Colorimetric Detection

**Figure 4:**
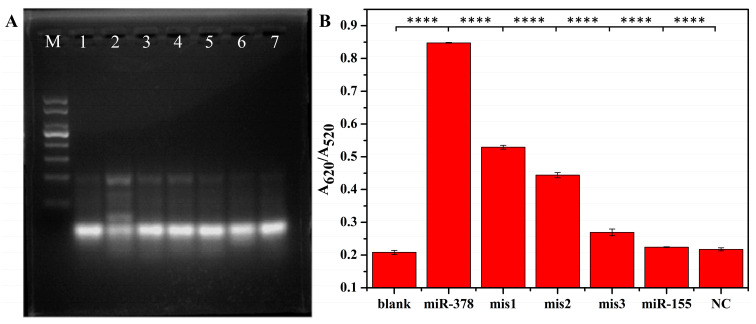
Specificity verification of colorimetric detection system. (**A**) Agarose gel electrophoresis verification. M: Marker; Lane 1: H1 + H2 + H3; Lane 2: H1 + H2 + H3 + miR-378; Lane 3: H1 + H2 + H3 + mis1; Lane 4: H1 + H2 + H3 + mis2; Lane 5: H1 + H2 + H3 + mis3; Lane 6: H1 + H2 + H3 + miR-155; and Lane 7: H1 + H2 + H3 + NC. (**B**) The specificity of colorimetric detection was verified using UV. ****: The signals of miR-378-containing groups were compared with those of other groups.

Figure 4 shows the selectivity of the developed system for detecting the agarose gel electrophoresis and UV absorption of mismatched one base (mis1), mismatched two bases (mis2), mismatched three bases (mis3), random sequence (NC), and the same concentration of miR-378. As shown in Figure 4A, in the presence of miR-378, lane 2 formed a brighter and slower migration band than that in the other groups, indicating that a Y-shaped structure was successfully formed in the presence of the target. Figure 4B shows that a strong detection signal was only generated when miR-378 was present, while the control sequence showed only a weak signal. This result proves that the method has a high specificity.

### 3.6. Sensitivity of Colorimetric Detection

**Figure 5:**
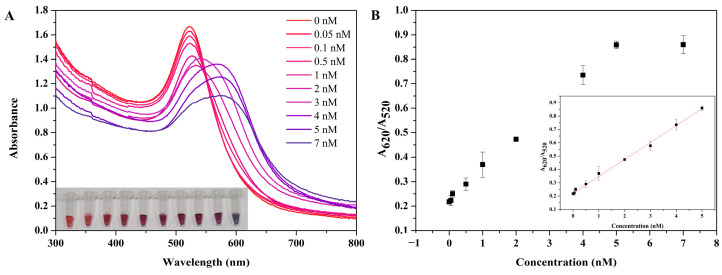
Study on the sensitivity of the colorimetric detection of miR-378. (**A**) UV-visible absorption spectra showing the colorimetric reaction of the detection system in the presence of different concentrations of target miR-378 and the image of AuNPs in the corresponding EP tubes. (**B**) The relationship between miR-378 concentration and relative absorbance (A_620_/A_520_). The error bar represents the standard deviation of three independent experiments.

To evaluate the role of the colorimetric biosensor detection target miR-378, the absorbance of miR-378 in the concentration range of 0.01 Nm–20 nM was measured using UV-visible technology. As shown in Figure 5A, with an increase in miR-378 concentration, the SPR absorption band of AuNPs in the 520 nm region decreased, and that in the 620 nm region increased. Meanwhile, the color of AuNP changed from red to purple. As shown in Figure 5B, the absorption ratio is linear in the range of 0.05–5 nM within the concentration range of miR-378. The minimum detection concentration of this method, at which color changes can be observed with the naked eye, is 0.05 nM, effectively demonstrating the capability of visual detection.

### 3.7. Biological Sample Application

**Figure 6:**
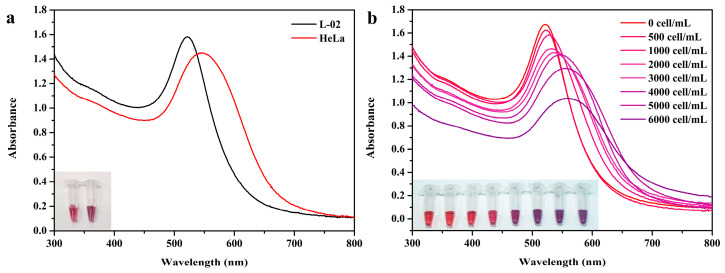
Study on the sensitivity of the colorimetric detection of total RNA in cell extracts. (**a**) UV results detected using L-02 cell and HeLa cell extracts and images of AuNPs in transparent EP tubes. (**b**) UV-visible absorption spectrum showing the colorimetric reaction of the detection system in the presence of different cell number extracts.

To verify the feasibility of this method for the determination of miRNAs in biological samples, we studied the detection of miR-378 in total RNA samples extracted from the cervical cancer cell line, HeLa, and the normal human liver cell line, L-02. The experimental results are shown in Figure 6. No obvious color change was observed in the system containing the L-02 extract. The maximum absorption peak of the system with HeLa extract was red-shifted, and the color changed from red to purple. Using HeLa cells for detection, the minimum number of cells that could be visually observed for a color change was 500 cells/mL.

## 4. Conclusions

In summary, a colorimetric biosensor based on AuNPs was constructed in this study. Three hairpins that could not only create a CHA reaction but also achieve the specific detection of miR-378 were designed. The functionalization of AuNPs was quickly achieved using a low-pH method containing a polyA fragment, which laid the foundation for subsequent colorimetric applications. On this basis, the target triggers the CHA so that a small amount of miR-378 can achieve a series of assembly reactions, realize the cyclic amplification of the detection, generate more Y-shaped structures, and shorten the distance between more nanoparticles, thus bringing about more color changes in the reaction system and providing visual detection. The colorimetric biosensor can be used to distinguish colors with the naked eye, with a minimum detection limit of 0.05 nM and good linearity at 0.05 nM–5 nM. The detection method is not only specific but also economical, convenient, and visual. This method is expected to be widely used for the visual analysis of tumor marker miRNAs.

## Data Availability

The original contributions presented in this study are included in the article/Supplementary Material. Further inquiries can be directed to the corresponding authors.

## Supplementary Materials

The following supporting information can be downloaded at https://www.mdpi.com/article/10.3390/bios15050319/s1. Figure S1: Secondary structure of hairpin H1, H2, H3; Figure S2: Optimization of detection conditions. a Poly A length optimization, including A10, A15 and A20. b Hybridization buffer pH optimization, including pH = 6, pH = 7, pH = 8 and pH = 9. c Optimization of the ratio of hairpin to gold nanoparticle concentration, including 50:1,75:1,100:1 and 125:1.; Table S1: DNA/RNA sequences used in experiments in this chapter; Table S2: Binding affinity data for complementary sequences used in the study.
