# Supplementary material for: Colorimetric Detection of microRNA-378 Based on Y-Shaped Structure Formed by Gold Nanoparticles and Catalytic Hairpin Self-Assembly
Source: Biosensors (Basel). 2025 May 15;15(5):319. doi: 10.3390/bios15050319 (PMC12110092; doi:10.3390/bios15050319)
Supplement: Supplementary file 1 [file biosensors-15-00319-s001.zip › biosensors-3579881-supplementary.pdf]

Supporting Information

Colorimetric detection of miR-378 based on Y-shaped structure formed by gold nanoparticles and catalytic hairpin self-assembly

Table S1 DNA /RNA sequences used in experiments in this chapter

Name	Sequence (5'-3')
H1-A10	A10-TGCCTTCTGACTCCAAGTCCAGTGAAGGAACACTGGACTTGGTGTCAG
H2-A10	A10-TAGATACCAAGTCCAGTGTTCCTTCTGGAGTCAGAAGGAACACTGGA
H3-A10	A10-CCAGTGTTCCTTCTGACTCCAACTGGACTTGGAGTCAGAAGGAAC
H1-A15	A15-TGCCTTCTGACTCCAAGTCCAGTGAAGGAACACTGGACTTGGTGTCAG
H2-A15	A15-TAGATACCAAGTCCAGTGTTCCTTCTGGAGTCAGAAGGAACACTGGA
H3-A15	A15-CCAGTGTTCCTTCTGACTCCAACTGGACTTGGAGTCAGAAGGAAC
H1-A20	A20-TGCCTTCTGACTCCAAGTCCAGTGAAGGAACACTGGACTTGGTGTCAG
H2-A20	A20-TAGATACCAAGTCCAGTGTTCCTTCTGGAGTCAGAAGGAACACTGGA
H3-A20	A20-CCAGTGTTCCTTCTGACTCCAACTGGACTTGGAGTCAGAAGGAAC
miR-378	ACUGGACUUGGAGUCAGAAGGC
Mis-1	ACUGGACUUGCAGUCAGAAGGC
Mis-2	ACUGGACAUGGAGUCACAAGGC
Mis-3	ACUGGACAUGGUGUCACAAGG
NC	UUGUACUACAAAAGUACUG
miR-155	UUAAUGCUAAUCGUGAUAGGGGU

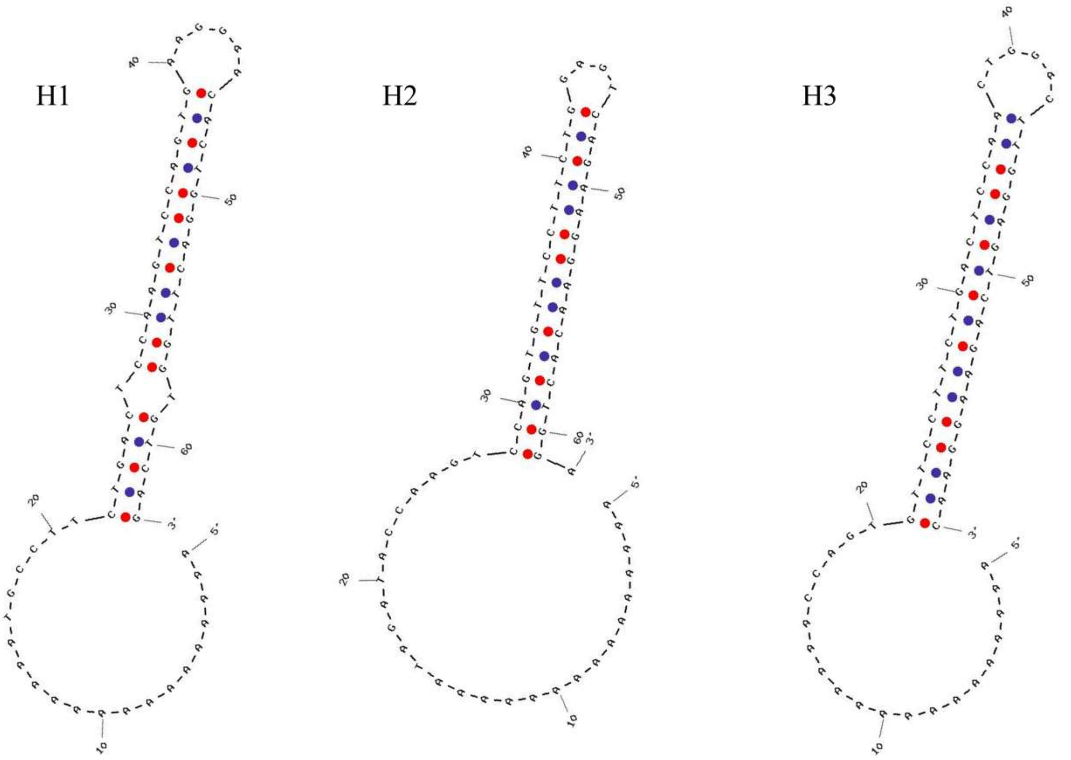

Figure S1 Secondary structure of hairpin H1, H2, H3

Table S2 Binding affinity data for complementary sequences used in the study

Name	Delta G
H1-miR-378	-40.42 kcal/mol
H1-H2	-35.79 kcal/mol
H2-H3	-37.63 kcal/mol
H3-H1	-37.28 kcal/mol

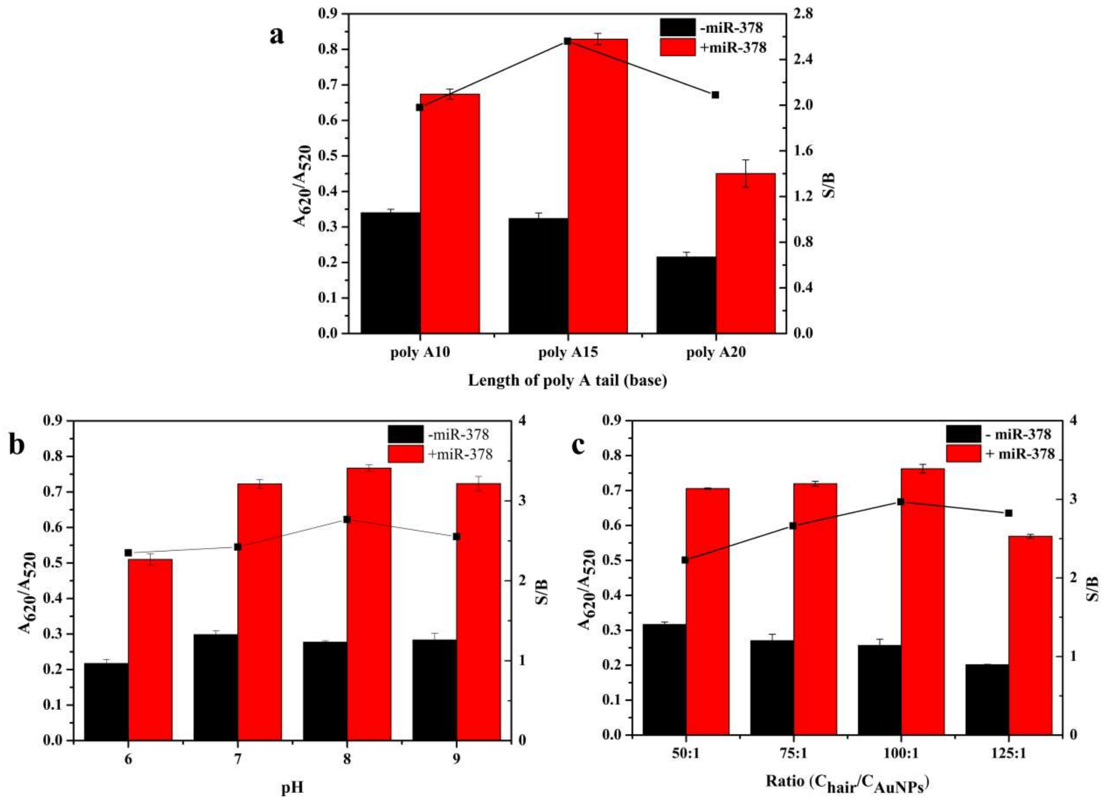

Figure S2 Optimization of detection conditions. a Poly A length optimization, including A10, A15 and A20. b Hybridization buffer pH optimization, including pH = 6, pH = 7, pH = 8 and pH = 9. c Optimization of the ratio of hairpin to gold nanoparticle concentration, including 50 : 1,75 : 1,100 : 1 and 125 : 1.
